# Vasoactive Effects of Chronic Treatment with ACE Inhibitor Zofenopril in Zucker Obese Diabetic Rats: The Role of Nitroso and Sulfide Signalization

**DOI:** 10.33549/physiolres.935722

**Published:** 2025-12-01

**Authors:** Ezgi SAMAN, Martina CEBOVA, Miroslava MAJZUNOVA, Andrea BERENYIOVA, Veronika GARAIOVA, Sona CACANYIOVA

**Affiliations:** 1 Institute of Normal and Pathological Physiology, Centre of Experimental Medicine, Slovak Academy of Sciences, Bratislava, Slovakia; 2 Institute of Pathophysiology, Faculty of Medicine, Comenius University, Bratislava, Slovakia; 3 Department of Animal Physiology and Ethology, Faculty of Natural Sciences, Comenius University, Bratislava, Slovakia

**Keywords:** Hydrogen sulfide, Nitric oxide, Obesity, Type 2 diabetes, Obese Zucker rats

## Abstract

Type 2 diabetes (T2D) associated with obesity is accompanied not only by metabolic but also cardiovascular disorders, including impaired vascular function. In addition to nitric oxide (NO), another gaseous transmitter, hydrogen sulfide (H_2_S), plays a key role in vascular homeostasis, but its function under pathological conditions is not fully understood. Escalated metabolic disorder associated with T2D could disrupt sulfide signaling and shift the balance between its pathological and compensatory action. The aim of the study was to investigate the role of H_2_S and NO signaling in the vascular function of obese Zucker diabetic fatty (ZDF) rats and to evaluate the impact of chronic treatment with zofenopril, an ACE inhibitor containing a sulfhydryl group. Cardiometabolic and biochemical parameters, as well as reactivity of the isolated thoracic aorta after 4 weeks of treatment, were assessed. Obese rats exhibited increased systolic blood pressure (SBP), cardiac and renal hypertrophy, increased adiposity, dyslipidemia, and impaired glucose tolerance compared with controls. Endothelium-dependent relaxation was reduced, with loss of H_2_S-derived relaxant component and dysregulation of NO signaling. Zofenopril significantly reduced SBP, attenuated cardiac and renal hypertrophy, and restored endothelial and contractile function. At the molecular level, it increased the expression of H_2_S-synthesizing enzymes, restored H_2_S-dependent vasorelaxation, and normalized NOS activity with a predominance of eNOS. In conclusion, zofenopril restored the balance of H_2_S and NO signaling in obese ZDF rats, thereby providing cardiovascular protection independent of improvements in glycemia or lipid profile. This dual mechanism may represent a promising therapeutic approach in preventing complications of obesity-induced T2D.

## Introduction

Cardiovascular complications in obesity-induced type 2 diabetes (T2D) derive from a complex interplay of endothelial dysfunction, oxidative stress, and dysregulated vascular signaling. In animal models, such as Zucker Diabetic Fatty (ZDF) rat, obesity and hyperglycemia lead to a significant impairment of endothelial function and nitric oxide (NO) signaling [1]. However, the changes in hydrogen sulfide (H_2_S) pathway can also play an important role in metabolic disorders. The reduced concentration of H_2_S in plasma has been confirmed in patients with T2D, whereby adiposity and obesity were determinants of this effect [2]. In healthy vasculature, endogenous H_2_S - largely derived from perivascular adipose tissue (PVAT) - provides anticontractile and anti-inflammatory support. In obesity, PVAT loses its ability to produce H_2_S and consequently fails to balance vascular tone [3]. On the other hand, the underlying mechanisms of sulfide signaling during pathological conditions are complex, as this pathway can also have a compensatory impact. H_2_S signaling was upregulated in normotensive rats fed fructose, female hypertriglyceridemic (HTG) rats, as well as in spontaneously hypertensive rats (SHR), where the increased participation of H_2_S in vasorelaxation compensated for disrupted angiotensin-converting enzyme 2 (ACE2) pathway [4, 5, 6]. We suppose that escalated metabolic disorder and obesity could significantly shift the balance between the pathological and compensatory H_2_S action.

Zofenopril, a sulfhydryl-containing ACE inhibitor, offers a dual mechanism of action: it attenuates angiotensin II-mediated vasoconstriction while releasing H_2_S with cardioprotective and anti-inflammatory effects [7]. H_2_S release helps to maintain the endothelial function beyond classical ACE inhibition, by restoring vasodilatory balance and mitigating redox imbalance [7, 8]. Moreover, our previous results confirmed that in hypertensive rats, zofenopril treatment increased the thoracic aorta vasorelaxation capacity due to stimulated participation of H_2_S and NO [9]. Despite these promising mechanisms, a gap remains in understanding how sustained zofenopril therapy could modulate H_2_S-dependent vascular signaling in the context of T2D. By addressing this gap, the present study examines the efficacy of 4 weeks of zofenopril treatment in decreasing vascular impairment in the obese ZDF rat model. We compared systolic blood pressure (SBP), biometric parameters, glucose utilization, endothelial function, and contractility of isolated thoracic aortas. The role of sulfide and nitroso signaling pathways was investigated by evaluating vasoactive responses, measuring protein expression of relevant enzymes, and determining NO synthase activity in aorta and H_2_S levels in plasma and heart.

## Materials and Methods

### Animals, ethical approval and the design of the study

Male Zucker Diabetic Fatty (ZDF) rats (fa/fa, obese phenotype) and lean heterozygous controls (fa/+) were obtained from the breeding station Dobrá Voda. The animals were bred in accordance with the institutional guidelines of the State Veterinary and Food Administration of the Slovak Republic and the Committee on the Ethics of Procedures in Animal, Clinical and other Biomedical Experiments (record number: 9704/2022) of the Centre of Experimental Medicine. All procedures complied with the European Convention for the Protection of Vertebrate Animals used for Experimental and other Scientific Purposes, Directive 2010/63/EU of the European Parliament. Animals were housed under controlled environmental conditions (22 ± 2 °C, 12-h light/dark cycle, relative humidity 50–60%), with ad libitum access to water.

15-week-old animals were randomly divided into three experimental groups (n = 8 per group): control lean fa/+ rats (L), obese fa/fa rats (O), and obese rats treated with zofenopril (OZ). Zofenopril calcium was administered p.o. at a dose of 15 mg/kg/day for 4 weeks, aged 16–20 weeks. Zofenopril was mixed with food, the amount of which was calculated based on food consumption to ensure daily intake. The study design included basal (at the 15^th^ week) and terminal (at the 20^th^ week) blood pressure monitoring, treatment administration (16^th^ – 19^th^week), intraperitoneal glucose tolerance test (IGTT, at the 20^th^ week), determination of plasma lipid profile (at the 20^th^ week), and terminal tissue collection for vascular reactivity study and biochemical analyses (at the 20^th^ week).

### Blood pressure measurements

SBP was recorded using a non-invasive tail-cuff plethysmography system (MRBP, IITC Life Science Inc., Los Angeles, CA, USA). Animals were trained for three consecutive days before baseline measurements to minimize stress-related variability. SBP readings were collected at baseline (15^th^ week, before beginning of zofenopril administration, basal SBP) and at the end of the 4-week treatment period (20^th^ week). Each reported value represents the mean of at least 5 stable measurements.

### Intraperitoneal glucose tolerance test

An IGTT was performed after the period of zofenopril treatment. Blood glucose was determined using a glucometer after an intraperitoneal administration of a 50% glucose solution load (2 mg/kg body weight) following overnight fasting. Blood was drawn from the tail before the glucose load at 0 min and thereafter at 30, 60, and 120 min.

### General biometric and plasma parameters

The body weight (BW) of each rat was determined before decapitation. The animals were sacrificed by decapitation after brief anesthetization with CO_2_. After decapitation, for the determination of the plasma parameters (glucose (GLU), total cholesterol (CHOL), high-density-lipoprotein-c cholesterol (HDL-C), triacylglycerols (TAG)), the trunk blood was collected into pre-prepared heparinized tubes (140 UI/5 ml) and then centrifuged (850xg, 10 min, 4°C, Centrifuge 5430 R, Eppendorf, Hamburg, Germany). The plasma samples were stored at 80°C until the selected parameters were measured. The levels of lipids were analyzed by a biochemical analyzer using auxiliary reagent discs (Celercare, MNCHIP Technologies Co., Ltd., Tianjin, China). 100 μL of plasma was pipetted into the sample chamber via the sample port. Then, 430 μL of distilled water was added to the diluent chamber via the diluent port of the test-specific reagent disk. The heart weight (HW), kidney weight (KDW), retroperitoneal fat weight (RFW) and tibia length (TL) were determined. The aliquots of tissue samples (heart, aortic arch, abdominal aorta) were removed, weighed, frozen in liquid nitrogen and stored at -80 °C for biochemical analysis. The thoracic aorta (TA) was isolated for further functional examination ex vivo.

### Vascular reactivity studies

The TA (descending part of the TA beginning below the aortic arch) was cleaned of connective tissue and cut into 5 mm long rings. The TA rings were vertically fixed between two stainless steel wire triangles and immersed in a 20 mL incubation organ bath with oxygenated (95% O_2_; 5% CO_2_) Krebs solution (118 mmol/lNaCl; 5 mmol/lKCl; 25 mmol/lNaHCO_3_; 1.2 mmol/lMgSO_4_.7H_2_O; 1.2 mmol/lKH_2_PO_4_; 2.5 mmol/lCaCl_2_; 11 mmol/l glucose; 0.032 mmol/lCaNa_2_EDTA) and kept at 37 °C. The upper wire triangles affixed to the TA ring were connected to isometric tension sensors (FSG-01, MDE, Budapest, Hungary), and changes in tension were registered by an NI USB-6221 AD converter (National Instruments, Austin, TX, USA and MDE, Budapest, Hungary) and S.P.E.L. Advanced Kymograph software (MDE, Budapest, Hungary). A resting tension of 1 g was applied to each ring and maintained throughout a 45- to 60-min equilibration period. Single concentrations of noradrenaline (10^−6^ mol/l) and acetylcholine (10^−5^ mol/l) were added to the organ bath to test the integrity of the arterial wall (the contractile ability and integrity of the endothelium). After washing with physiological Krebs solution and an equilibration period, experiments with NA were started to obtain contractile responses. Adrenergic contractions were determined in the TA as the responses to cumulatively applied exogenous noradrenaline. The contractile responses were expressed as the active wall tension in grams and normalized to the length of the respective ring preparation (mm). To examine endothelium-dependent vasorelaxation, increasing concentrations of acetylcholine were applied in a cumulative manner to noradrenaline- precontracted aortic rings. The rate of relaxation was expressed as a percentage of the maximum noradrenaline-induced contraction.

Next, we examined the participation of certain signaling pathways in the vasoactive responses of the TA. To determine the role of the endogenous NO pathway, the rings of the TA were incubated with a nonspecific inhibitor of NO synthase, NG-nitro-L-arginine methyl ester (LN, 10^−5^ mol/l). The H_2_S scavenger bismuth (III) subsalicylate (BSC, 10^−5^ mol/l) was used to evaluate the participation of H_2_S in the vasoactive responses. To determine the effects of the inhibitors on contractile responses and endothelium-derived relaxation, all the mentioned compounds were acutely incubated for 20 min in an organ bath, and the concentration–response curves to noradrenaline and acetylcholine were repeated.

### Total NO-synthase activity

Total NO-synthase (NOS) activity was determined in crude homogenates of the aorta by measuring the formation of [^3^H]-L-citrulline from [^3^H]-L-arginine (ARC, St. Louis, MO, USA) as previously described and slightly modified by Pechanova *et al.* [10]. [^3^H]-L-Citrulline was measured with the Quanta Smart TriCarb Liquid Scintillation Analyzer (Packard Instrument Company, Meriden, CT). NOS activity was then normalized to protein content and expressed as picokatal per gram of protein (pkat/g protein).

### Western blotting

Aortic samples were homogenized on ice in 0.05 mol/l Tris buffer (pH 7.4) supplemented with protease inhibitors. The protein concentrations were determined via a Lowry assay. Proteins (20 μg total protein) were separated by 12% or 15% SDS-PAGE depending on the size of the protein being measured and transferred to nitrocellulose membranes. The membranes were blocked with 5% milk in Tris-buffered saline containing Tween 20 (TBS-T). Afterward, the membranes were incubated with a rabbit polyclonal anti-eNOS antibody (Abcam, Cambridge, UK; dilution 1:1000), a rabbit polyclonal anti-iNOS antibody (Proteintech®, Manchester, UK; dilution 1:1000), a rabbit polyclonal anti-CBS antibody (Proteintech®, Manchester, UK; dilution 1:3000), a mouse monoclonal anti-CSE antibody (Proteintech®, Manchester, UK; dilution 1:5000) overnight at 4 °C. All the blots were reprobed with a rabbit polyclonal anti-β-actin antibody (Abcam, Cambridge, UK; dilution 1:5000) overnight at 4 °C. The membranes were incubated with anti-rabbit (Abcam, Cambridge, UK) or anti-mouse (Cell Signaling, Danvers, MA, USA) secondary peroxidase-conjugated antibodies at room temperature for 2 h. Both the primary and secondary antibodies were diluted in TBS-T containing 1% milk. The signals were visualized with Clarity Western ECL Substrate (Bio-Rad, Inc., Hercules, CA, USA) *via* ChemiDocTM Touch Imaging System (Bio-Rad) and quantified with Image Lab Software. The target protein amounts were normalized to those of β-actin and are presented in arbitrary units (a.u.).

### Determination of H_2_S concentration in plasma and heart tissue

The concentration of H_2_S in plasma was assessed using the methylene blue assay, as described previously with slight modifications [11]. The essential points are as follows: 100 μl of plasma and 400 μl 0.1M potassium phosphate buffer (PPB, pH 7.4) were combined. The reaction mixture was prepared by mixing zinc acetate (250 μl, 1%), trichloroacetic acid (250 μl 10%; TCA), N, N-dimethyl-p-phenylenediamine sulphate (133 μl, 20 mmol/l in 7.2 mol/l HCl; DPD), and iron (III) chloride (133 μl, 30 mmol/l in 1.2 M HCl; FeCl_3_) [12]. After 25 minutes of incubation at room temperature in a dark environment, all samples and standards were centrifuged at 12.000 rpm at 4 °C for 5 minutes. Supernatants were transferred in duplicate to a 96-well plate. The absorbance of the final solution was measured at 630 nm using a spectrophotometer (NanoDrop^TM^ 2000/2000c Spectro-photometers, Thermo Fisher Scientific, Waltham, MA, USA). Sodium sulfide (Na_2_S) was utilized to create a calibration curve, and H_2_S levels were calculated against a calibration curve ranging from 3.9 μmol/l to 250 μmol/l of Na_2_S with a blank. Plasma H_2_S concentrations are indicated as μmol/l.

The homogenization of the heart tissue was performed using a lysis buffer containing sodium orthovanadate and protease inhibitor. Total protein concentrations were quantified using the Lowry protein assay. To standardize protein content, homogenates were adjusted to equal amounts of protein (25 μg) [4]. The reaction mixture was prepared with homogenates (25 μg), PPB (0.1 mol/l), saline (0.9%, 30 μl), and pyridoxal 5’-phosphate (30 μl, 2 mmol/l; PP). Samples designated for basal H_2_S production contained only PPB. To trigger the reaction, samples were incubated in a 37 °C water bath for 30 minutes in a dark environment. The following steps were performed, including measurement and calculation as described in the plasma H_2_S measurement protocol.

### Immunofluorescent detection of enzymes

Cryosections of the thoracic aorta (embedded in Tissue-Tek O.C.T. compound, Leica Biosystems, Deer Park, IL, USA) with a thickness of 7 μm were prepared for immunofluorescence staining to assess the localization of CSE and CBS enzymes. Sections were mounted on Superfrost Plus adhesion slides (Epredia™, Fisher Scientific, Waltham, MA, USA) and fixed with 4% paraformaldehyde. To reduce background fluorescence, slides were treated with 50 mM NH_4_Cl, permeabilized with 0.25% Triton X-100, and subsequently blocked with 5% goat serum in phosphate-buffered saline (PBS, pH 7.4). Primary antibodies against CSE and CBS (Proteintech, Manchester, UK; diluted 1:100 in 1% goat serum) were applied overnight at 4 °C. After washing, appropriate fluorescent secondary antibodies were added: FITC-conjugated antibody for CBS (Abcam, Cambridge, UK; diluted 1:2000) and Alexa Fluor 532-conjugated antibody for CSE (Thermo Fisher Scientific, Waltham, MA, USA; diluted 1:2000). Nuclear counterstaining was performed with DAPI-containing Vectashield antifade medium (Vector Laboratories Inc., Burlingame, CA, USA). Images were acquired using a Cytation 5 imaging reader (BioTek, Winooski, VT, USA) and processed with Gen5 software.

### Statistical analysis

The group size was calculated via a priori analysis via G*Power software v3.1, and the total sample size was calculated to be 32 (n = 8/group). The normality of the data was tested via the Shapiro-Wilk test. Data are presented as mean ± SEM. Between-group differences were analyzed using one-way, two-way, or three-way ANOVA where appropriate, followed by Bonferroni post hoc tests. A p-value < 0.05 was considered statistically significant. Data are expressed as the means ± S.E.M.s and were analyzed via OriginPro (OriginLab Corporation, Northampton, MA, USA).

### Drugs

The following drugs were used: acetylcholine, bismuth (III) subsalicylate, N^G^-nitro-L-arginine methyl ester, all from Merck (Darmstadt, Germany), zofenopril calcium from AdooQ Bioscience (Irvine, CA, USA), and noradrenaline from Zentiva (Prague, Czech Republic).

## Results

Basic biometric data and the level of selected plasma parameters in individual groups at the end of the treatment are shown in Table 1. Obese ZDF rats had markedly higher BW than lean controls (p < 0.001), and zofenopril treatment did not significantly affect this. Retroperitoneal fat mass, absolute weight - RFW, as well as in relation to tibia length (RFW/TL), was also markedly elevated in obese rats (both p < 0.001), and zofenopril treatment did not affect the adiposity significantly. Obese rats showed mild but significant cardiac hypertrophy, evidenced by increased HW and HW/TL ratio (both p < 0.05). Zofenopril treatment reversed and normalized cardiac hypertrophy in the treated group (p < 0.01). Similarly, renal hypertrophy was observed in obese rats as reflected by increased absolute weight - KW and KDW/TL ratio (both p < 0.001). Both indices were significantly reduced by zofenopril treatment (p < 0.01; p < 0.001), suggesting renoprotective effects.

Plasma biochemical profiles provided additional confirmation of the obese phenotype. GLU changes did not differ significantly among all the groups. TAG levels were significantly elevated in obese rats (p < 0.001); however, treatment with zofenopril did not reduce them; instead, TAG levels were slightly higher than in obese rats (p < 0.05). Total CHOL levels were significantly higher in obese rats (p < 0.01). Zofenopril did not indicate a lipid-lowering effect since it did not decrease the CHOL level.

Similarly, HDL-C levels were increased in both groups of obese rats regardless of zofenopril treatment (both p < 0.001), with no significant difference between the obese and the treatment groups. The levels of H_2_S in plasma and the heart were significantly decreased in obese rats (both p < 0.05). Zofenopril treatment increased H_2_S levels (both p < 0.05) and returned them to the level of control lean rats (Table 1).

**Fig. 1:**
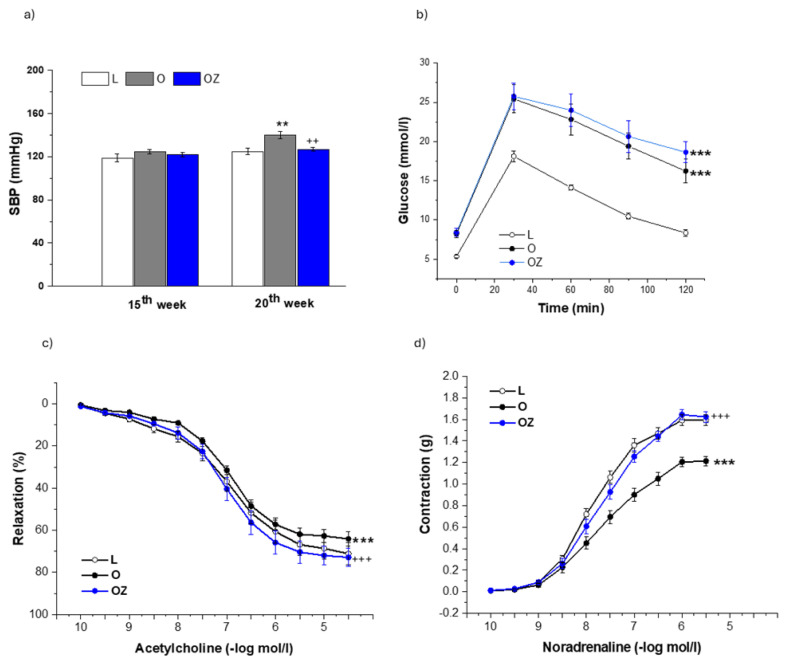
Systolic blood pressure (SBP) (**a**), glucose utilization (**b**), endothelium dependent vasorelaxation of the isolated thoracic aorta (**c**), and noradrenaline-induced contraction of the isolated thoracic aorta in control lean (L, n=8), obese (O, n=8), and zofenopril-treated obese (OZ, n=8) rats (**d**). SBP was evaluated before the beginning of zofenopril administration (15^th^ week) and at the end of the 4-week treatment period (20^th^ week). An intraperitoneal glucose tolerance test was accomplished at the end of the treatment with zofenopril. The results are presented as mean ± S.E.M., and differences among groups were analyzed by one-way (a) or two-way (b,c,d) ANOVA, **p<0.01, ***p<0.001 vs L; ^++^ p<0.01, ^+++^ p<0.001 vs O.

At the end of the experiment, SBP was significantly elevated in obese compared with lean rats (p<0.01). After 4 weeks of treatment, zofenopril significantly reduced SBP in obese rats (p < 0.01; Fig. 1a). The IGTT revealed impaired glucose tolerance in obese rats, with elevated glucose levels at all post-load time points (F = 70.86; p = 4.79 x 10^−21^), which was also confirmed by the Bonferroni post hoc test (p < 0.001). Zofenopril treatment did not significantly modify glucose intolerance within the 4-week treatment period, suggesting its vascular benefits occur independently of systemic glucose control (Fig. 1b).

**Fig. 2:**
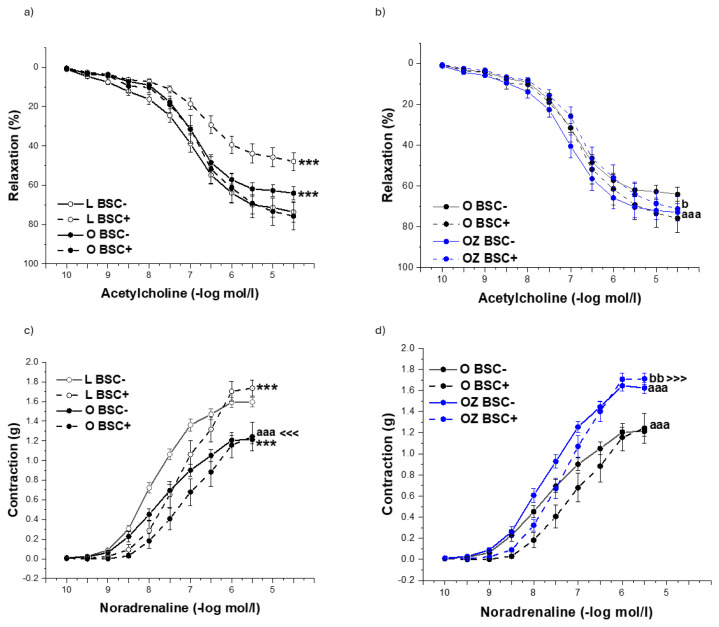
The role of H_2_S signaling in endothelium-dependent vasorelaxation (**a,b**) and adrenergic contraction (**c,d**) of the isolated thoracic aorta in control lean (L, n=8), obese (O, n=8), and zofenopril-treated obese (OZ, n=8) rats. BSC- represents the rings without the incubation with H_2_S scavenger bismuth (III) subsalicylate, BSC+ represents the rings with the incubation with H_2_S scavenger bismuth (III) subsalicylate. The results are presented as mean ± S.E.M., and differences among groups were analyzed by three-way ANOVA, *** p<0.001 vs L BSC-; ^<<<^ p<0.001 vs L BSC+; aaa p<0.001 vs O BSC-; ^>>>^ p<0.001 vs O BSC+; b p<0.05, bb p<0.01 vs OZ BSC-.

**Fig. 3:**
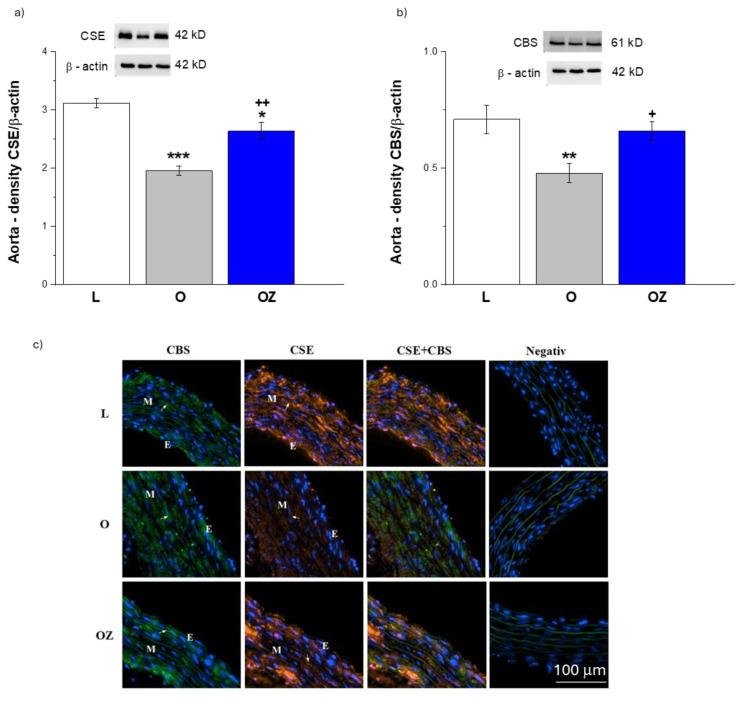
The protein expressions of H_2_S-producing enzymes – cystathionine γ-lyase (CSE) (**a**), cystathionine β-synthase (CBS) (**b**) – and their distribution (CBS – green, CSE - orange) (**c**) in the thoracic aorta in control lean (L, n=8), obese (O, n=8), and zofenopril-treated obese (OZ, n=8) rats. E - endothelium, M - tunica media. Blue fluorescence indicates cell nuclei stained with 4′,6′-diamidino-2-phenylindole (DAPI). Images captured at 20× magnification. The results are presented as mean ± S.E.M., and differences among groups were analyzed by one-way ANOVA, *p<0.05, **p<0.01, ***p<0.001 vs L; ^+^ p<0.5, ^++^ p<0.01 vs O.

Evaluation of endothelium-dependent relaxation responses confirmed the impact of both obesity and zofenopril administration on endothelial function (F = 11.54, p = 1.28 x 10^−5^, Fig. 1c). Obese ZDF rats demonstrated significantly reduced acetylcholine-induced endothelium-dependent relaxation compared with lean controls (p < 0.001). After 4 weeks of treatment with zofenopril, the rats exhibited significantly improved relaxation (p < 0.001), approaching values observed in lean controls. We similarly confirmed the effect of obesity and the zofenopril treatment on aortic contractility (F = 80.57, p = 1.54 x 10^−30^, Fig. 1d). We observed significantly decreased dose-dependent contractile responses to noradrenaline in obese rats (p < 0.001); however, the treatment with zofenopril reversed this effect (p < 0.001). Evaluation of endothelium-dependent relaxation responses confirmed the participation of endogenous H_2_S in the control of endothelial function in lean rats predominantly (F = 61.05, p = 4.06 x 10^−14^, Fig. 2a). In lean control rats, acetylcholine-induced vasorelaxation responses were attenuated by the pretreatment with H_2_S scavenger bismuth (III) subsalicylate (BSC, 10^−5^ mol/l, p < 0.001), indicating that endogenous H_2_S contributes to the maintenance of endothelial function *via* supporting vasodilatory action under physiological conditions. In contrast, the pretreatment with BSC showed a non-significant effect in obese rats, suggesting that H_2_S contribution to endothelial relaxation was diminished in obesity (Fig. 2a). On the other hand, administration of zofenopril refreshed the participation of endogenous H_2_S in maintaining endothelial function since the pretreatment with BSC reduced acetylcholine-induced relaxation in obese rats treated with zofenopril (Fig. 2b), indicating restoration of the H_2_S-dependent part of vasorelaxation. Regarding the contractility, we confirmed the participation of endogenous H_2_S in both lean and obese rats (F = 39.61, p = 7.66 x 10^−10^, Fig. 3c), where in both groups it had a pro-contractile effect (both p < 0.001). Administration of zofenopril increased the noradrenaline-induced contractile response in obese rats (p < 0.001), and endogenously produced H_2_S contributed to this increase (p < 0.01, Fig. 2d).

H_2_S-producing enzyme expressions in the aorta are shown in Fig. 3. Compared with lean controls, CSE protein levels were significantly reduced in the obese group (p < 0.001). Zofenopril treatment markedly increased CSE expression (p < 0.01); the recovery appeared partial for CSE as the expression remained still reduced compared to the control group (p < 0.05; Fig. 3a). Similarly, CBS protein levels were also significantly decreased in obese rats compared with lean controls (p < 0.01). Treatment with zofenopril notably elevated CBS expression (p < 0.05), with the recovery appearing closer to lean controls (Fig. 3b). Fig. 3c is an illustrative topography of H_2_S-producing enzymes in the thoracic aorta. Fluorescent staining indicates greater amounts of CSE and CBS enzymes in obese rats after zofenopril administration.

**Fig. 4:**
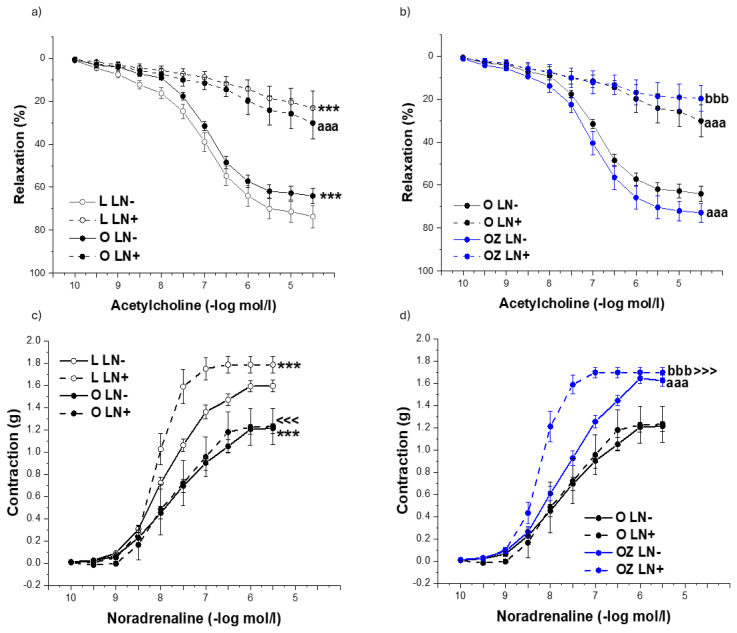
The role of NO signaling in endothelium-dependent vasorelaxation (**a,b**) and adrenergic contraction (**c,d**) of the isolated thoracic aorta in control lean (L, n=8), obese (O, n=8), and zofenopril-treated obese (OZ, n=8) rats. LN- represents the rings without the incubation with NO inhibitor NG-Nitro-L-arginine methyl ester, LN+ represents the rings with the incubation with NO inhibitor NG-Nitro-L-arginine methyl ester. The results are presented as mean ± S.E.M., and differences among groups were analyzed by three-way ANOVA, *** p<0.001 vs L LN-; ^<<<^ p<0.001 vs L LN+; aaa p<0.001 vs O LN-; ^>>>^ p<0.001 vs O LN+; bbb p<0.001 vs OZ LN-.

**Fig. 5:**
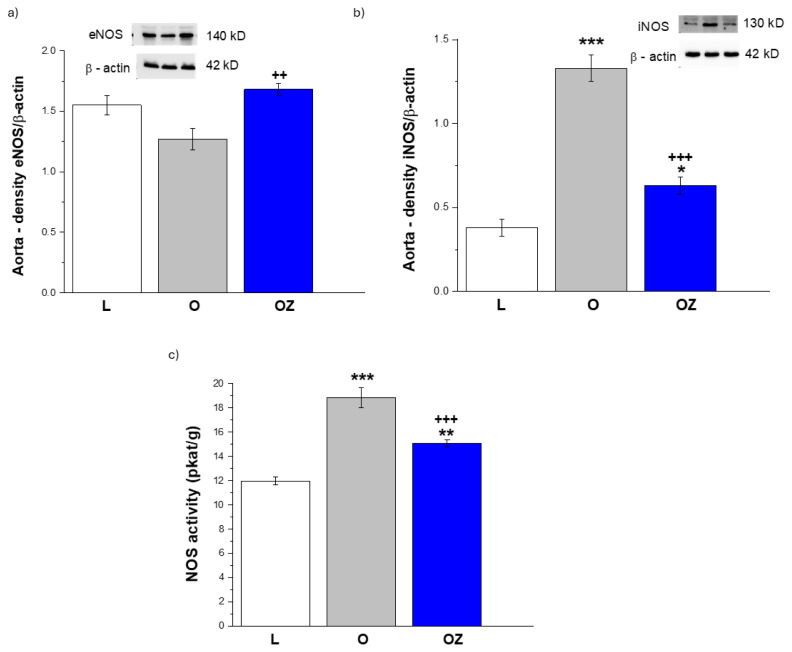
The protein expressions of NO-producing enzymes – endothelial nitric oxide synthase (eNOS) (**a**) and inducible nitric oxide synthase (iNOS) (**b**), and total nitric oxide synthase activity (NOS, pkat/g) (**c**) of the thoracic aorta in control lean (L, n=8), obese (O, n=8), and zofenopril-treated obese (OZ, n=8) rats. The results are presented as mean ± S.E.M., and differences among groups were analyzed by one-way ANOVA, *p<0.05, **p<0.01, ***p<0.001 vs L; ^++^ p<0.01, ^++ +^p<0.001 vs O.

Experiments with NO synthase inhibitor, N^G^-nitro-L-arginine methyl ester (LN,10^−5^ mol/l) confirmed that the acute pre-incubation of aortic rings with LN significantly inhibited vasorelaxant responses in both lean (p < 0.001) and obese (p < 0.001) rats confirming the role of NO in endothelial function of healthy and obese rats (F = 471.37, p → 0, Fig. 4a). The impact of zofenopril treatment on vasorelaxation response is shown in Fig. 5b. The inhibition of acetylcholine-induced relaxation after acute administration of LN was similar in treated and untreated obese rats (both p < 0.001) (Fig. 4b). However, since the relaxation response in the absence of inhibitor was greater after zofenopril administration than in obese rats, the NO component probably increased by the treatment. Concerning the effect of noradrenaline-induced contraction, pre-treatment with LN significantly increased contractile responses in lean rats only (F = 16.04, p = 7.29 x 10^−5^, Fig. 4c). In obese rats, this LN-induced enhancement of contraction was diminished, indicating a loss of NO-dependent modulation of arterial tone (Fig. 5c). Zofenopril treatment restored the effect of LN, with a significant increase in noradrenaline-induced contraction compared to untreated obese rats, demonstrating recovery of the NO-mediated regulatory mechanism (p < 0.001, Fig. 4d).

The expression of NOS enzymes in aortic tissue is presented in Fig. 5. Endothelial eNOS protein levels were slightly lower in obese rats when compared to lean controls; however, the difference was not significant. While treatment with zofenopril significantly elevated eNOS expression (p < 0.01, Fig. 5a). In contrast, as related to pro-inflammatory NOS isoform, iNOS protein levels were markedly increased in obese rats compared with lean animals (p < 0.001, Fig. 5b). Furthermore, a significant reduction of elevated iNOS levels was observed after the zofenopril treatment (p < 0.001, Fig. 5b).

Consistent with these findings, total NOS activity was significantly increased in obese rats relative to lean controls (p < 0.001, Fig. 5c), largely reflecting iNOS upregulation. Zofenopril administration showed a reduction in total NOS activity compared to untreated obese rats (p < 0.001, Fig. 5c). This finding indicates the normalization of NOS activity through suppression of iNOS and preservation of eNOS.

## Discussion

In our study we proved that obese rats displayed significantly higher SBP and cardiac hypertrophy compared with lean controls, reflecting the well-established link between obesity, hypertension and cardiac remodeling. Underlying mechanisms has been reported as increased sympathetic activity, renin-angiotensin-aldosterone system activation, impaired natriuretic signaling, inflammation, and dysfunction of PVAT, which together drive hypertrophy and fibrosis [13, 14]. In relation to therapy we showed that four weeks of zofenopril treatment significantly lowered SBP and normalized cardiac remodeling. Comparable alteration has also been reported in the model of myocardial ischemia/reperfusion injury where the treatment with zofenopril protected the myocardium from remodeling by increasing the bioavailability of NO and H_2_S [15]. Consistent with the dual mechanism of action [16] – classical ACE inhibition and increasing H_2_S availability, which was also confirmed by our results – zofenopril probably contributed to cardioprotection independent of the net decrease in SBP. Moreover, our results also showed that obese rats developed renal hypertrophy, which reflects one of early signs of diabetic kidney nephropathy [17]. With zofenopril treatment, renal hypertrophy was reduced, showing its protective effect on the kidneys in the setting of obesity and diabetes. This agrees with evidence that sulfhydryl ACE inhibitors not only lower SBP but also improve oxidative balance and slow the development of renal injury [16].

We proved that obesity in ZDF rats was associated with clear dyslipidemia, as shown by elevated TAG and CHOL, along with greater adiposity and glucose intolerance. These findings agree with previous studies, which describe elevated serum lipids, accumulation of free fatty acids, and adipocyte hypertrophy in obese models, including high-fat diet-induced obesity [18], human obesity [19], and ZDF rats [20]. In our study, zofenopril did not improve lipid parameters; indeed, TAG levels were slightly higher after treatment. This contrasts with some clinical studies that reported beneficial effects of zofenopril on lipid profiles in patients with metabolic syndrome [21]. Discrepancy may reflect differences between species, as ZDF rats carry a leptin receptor mutation [22], whereas in humans, obesity is usually diet-induced. However, an earlier clinical study in non-diabetic patients with mild to moderate essential hypertension reported no modification of lipid profile after 12-week lasting zofenopril treatment, indicating its inconsistent effects [23].

In obese rats, we showed an impairment of endothelial function and vascular hypocontractility, while zofenopril treatment improved acetylcholine-mediated relaxation and normalized responses to noradrenaline. Previous studies pointed out that the age and/or sex of models could influence the vascular dysfunction. In ZDF rats, acetylcholine-induced relaxation was found to be impaired in females at 16 weeks [1], and unchanged or reduced up to 24 weeks in males [24]. Our results showed a slight but significant impairment of endothelial function at 20 weeks, confirming that endothelial dysfunction in ZDF rats progresses with T2D. In terms of contractility, obese rats displayed decreased noradrenaline-induced contractions, consistent with altered vascular reactivity in obesity. Oltman *et al.* [24] found attenuated adrenergic responses in ZDF rat aortas at 8–12 weeks, suggesting early development of dysfunction. Possible causes of the altered contractile properties include receptor desensitization or changes in calcium sensitivity/handling. One factor may be inflammation as supported by increased iNOS expression in this study. Trovato *et al.* [25] showed that obesity-related metabolic disorders can impair muscle metabolism, leading to muscle damage associated with elevated inflammatory factors. Zofenopril treatment normalized adrenergic vasocontraction indicating restoration of vascular tone regulation, which was accompanied by decreased iNOS protein expression. However, to confirm the role of inflammation or its suppression after zofenopril administration, it would be necessary to assess additional inflammatory markers. Another possible explanation is the specific pharmacological profile of zofenopril as an H_2_S donor. This additional mechanism may influence the complex H_2_S–NO interactions, leading to more pronounced effects on the regulation of contractility and vascular function in general.

Our previous studies across several models have demonstrated that H_2_S acted as a compensatory signal under the conditions of vascular stress, such as hypertension, hyperglycemia or metabolic imbalance. This was confirmed in fructose and ACE2-inhibitor treated SHR, prediabetic male and female rats or in human intrarenal arteries of hypertensive patients [9, 26, 27, 28]. In contrast, our current results in obese ZDF rats demonstrated reduced H_2_S levels in both plasma and heart tissue, which was linked to the loss of H_2_S-dependent component of vasorelaxation. Moreover, while the procontractile effect of H_2_S could reflect an attempt to compensate for reduced adrenergic contraction in obese rats, endogenous sulfide signaling is generally considered beneficial due to its anticontractile effect, as observed in SHR and non-obese HTG rats. [28,29]. Taken together severe metabolic disturbances in obesity weaken compensatory action of H_2_S, with obesity itself probably being the main limiting factor. Katsouda *et al.* [30] similarly reported downregulation of CSE, CBS, and 3-mercaptopyruvate sulfurtransferase (3-MST) with reduced H_2_S in adipose depots of mice fed a Western diet, while Candela *et al.* [31] demonstrated that microvascular endothelial dysfunction in obesity was driven by macrophage-dependent depletion of H_2_S in vessels which was reversed by restoring H_2_S availability. Moreover, we also observed NO pathway dysregulation in obese ZDF rats. Although total NOS activity increased, it was mainly due to elevated iNOS expression, indicating shift in NO signaling leading to reduced bioavailability from nitrosative stress. This aligns with previous diabetic and obese models showing eNOS uncoupling, increased iNOS, and reactive oxygen species-mediated disruption of vascular NO signaling, with Nox2-derived superoxide and peroxynitrite formation contributing to endothelial dysfunction [32,33]. After four weeks of treatment, zofenopril improved both H_2_S and NO pathways by increasing H_2_S levels and the expression of H_2_S-synthesizing enzymes (CSE, CBS), restoring the H_2_S-dependent component of vasorelaxation, and transforming NOS activity toward eNOS. These findings agree with evidence that zofenopril and its active metabolite zofenoprilat enhance H_2_S availability and expression of its producing enzymes, providing vascular protection beyond ACE inhibition. Several studies showed that zofenopril restored H_2_S levels and improved vascular relaxation [34], stimulated H_2_S production, Akt/eNOS/ERK signaling in endothelial cells and angiogenesis [35], and increased NO and H_2_S bioavailability to reduce myocardial injury [15].

Despite our solid findings, this study has certain limitations. The intervention period was restricted to four weeks, which may not reflect the long-term effects of zofenopril. Although the ZDF rat is a well-established model of obesity-associated diabetes, its leptin receptor mutation might not fully represent the multifactorial origins of human obesity. In addition, key metabolic parameters such as insulin sensitivity and detailed lipid subfractions were not evaluated. Future research should take it all into account and address translational potential in clinical settings.

The present study demonstrated that obesity and T2D were associated with cardiovascular and metabolic disorders, including dyslipidemia, impaired glucose tolerance, elevated SBP as well as endothelial dysfunction and impaired contractility associated with loss of endogenous H_2_S support, and dysregulation of NO signaling. Zofenopril treatment improved endothelial function and contractility, reactivated H_2_S pathway and restored NO balance providing cardiovascular protection independent of metabolic correction of glycemia or lipid metabolism. Zofenopril’s benefits were mainly vascular and organ-protective and support its use in obesity- and diabetes-induced vascular disorders.

## Figures and Tables

**Table 1:** The basic biometric parameters and plasma lipid profile in ZDF lean, obese, and obese rats treated with zofenopril at the end of the experiment

Parameter	L	O	OZ
*n*	8	8	8
*BW (g)*	381 ±10.30	548.13 ±12.6***	523.38 ±10.3***
*RFW (g)*	1.503 ±0.16	11.30 ±0.33***	11.30 ±0.39***
*HW (g)*	1.265 ±0.056	1.447 ±0.035*	1.283 ±0.032++
*KDW (G)*	1.137 ±0.053	1.43513 ±0.046***	1.21 ±0.032++
*TL (mm)*	35.11 ±0.329	34.3 ±0.303	34.15 ±0.36
*RFW/TL (mg/mm)*	42.98 ±4.999	329.4 ±9.248***	331.4 ±13.35***
*HW/TL (mg/mm)*	36 ±1.396	42.22 ±1.146**	37.53 ±0.678++
*KDW/TL*	32.35 ±1.374	41.84 ±1.285***	35.4 ±0.641+++
*GLU (g/l)*	9.345 ±0.316	11.98 ±2.632	9.542 ±0.559
*TAG (mmol/l)*	1.181 ±0.089	8.309 ±0.739***	9.587 ±0.048***+
*CHOL (mmol/l)*	3.006 ±0.058	5.85 ±0.542**	5.135 ±0.306***
*HDL-C (mmol/l)*	1.785 ±0.039	2.589 ±0.149***	2.64 ±0.121***
*H* *_2_* *S plasma (μmol/l)*	40.96±2.09	34.33±1.99*	40.96±2.18+
*H* *_2_* *S heart (μmol/l)*	42.72±3.61	32.79±2.23*	43.07±3.79+

Abbreviations: L – lean ZDF rats, O – obese ZDF rats, OZ - obese rats treated with zofenopril. BW – body weight, HW – heart weight, TL – tibia length, HW/TL – ratio of heart weight to tibia length, KDW – kidney weight, KDW/TL – ratio of kidney weight to tibia length, RFW – weight of retroperitoneal fat, RFW/TL - ratio of weight of retroperitoneal fat to tibia length, CHOL – total cholesterol, HDL-C- high-density lipoprotein cholesterol, TAG – triacylglycerols, n – number of rats. The results are presented as mean ± S.E.M., and differences among groups were analyzed by one-way ANOVA;

* p<0.05

** p<0.01;

*** p<0.001vs L;

+ p<0.05vs O;

++ p<0.01vs O;

+++ p<0.001vs O.
